# Bats, Coronaviruses, and Deforestation: Toward the Emergence of Novel Infectious Diseases?

**DOI:** 10.3389/fmicb.2018.00702

**Published:** 2018-04-11

**Authors:** Aneta Afelt, Roger Frutos, Christian Devaux

**Affiliations:** ^1^Interdisciplinary Center for Mathematical and Computational Modelling, University of Warsaw, Warsaw, Poland; ^2^IES, Univ. Montpellier, CNRS, Montpellier, France; ^3^Cirad, UMR 17, Intertryp, Montpellier, France; ^4^Aix Marseille Université, Centre National de la Recherche Scientifique, IRD, Institut National de la Santé et de la Recherche Médicale, AP-HM, URMITE, IHU-Méditerranée Infection, Marseille, France

**Keywords:** bat, coronavirus, deforestation, emergence, anthropization, novel contacts, mosaic landscape

## Introduction

Coronaviruses (CoV) were for a long time associated with several major veterinary diseases such as avian infectious coronavirus, calf diarrhea, winter dysentery, respiratory infections (BRD-BCoV) in cattle, SDCV, PEDV, SECD in swine and dog, intestinal disease or Feline Infectious Peritonitis (Saif, 2014), and the human mild and common cold. However, SARS emerged in 2002 in China and spread across 29 other countries with a 10% death rate. More recently, the MERS-CoV outbreak in Saudi Arabia in 2012 displayed a death rate of 38%. The emergence of these two events of highly pathogenic CoVs shed light on the threat posed by coronaviruses to humans. Bats are hosting many viruses (Calisher et al., 2006) and in particular coronaviruses, which represent 31% of their virome (Chen et al., 2014). Furthermore, bats display a remarkable resistance to viruses (Omatsu et al., 2007; Storm et al., 2018). The risk of emergence of a novel bat-CoV disease can therefore be envisioned.

## Of bats and men

Although human blood has been found in the diet of *D. ecaudata* bats in Brazil (Ito et al., 2016), indicating that bats can feed on humans, this is exceptional. Furthermore, with perhaps the exception of Australian Bat Lyssavirus (ABLV) and Duvanhage virus, there is no clear case of direct transmission of the virus from bats to humans (Tignor et al., 1977; Hanna et al., 2000; Paweska et al., 2006). Usually, bats are beneficial to humans by playing a major role in agriculture since they pollinate fruit trees (Whittnaker et al., 1992; Kelm et al., 2008) and help controlling populations of insects (Leelapaibul et al., 2005; Kalka et al., 2008). Today, in Asia, 56 species of bats are hunted and consumed by low-income populations (Mildenstein et al., 2016). They are also used in traditional medicine (Walker, 2005; Ashwell and Walston, 2008) and on farms for the production of guano (Chhay, 2012; Thi et al., 2014). Bioinformatic analysis suggested that there were already several CoV transmission events between bats, civets and humans before the 2002 SARS outbreak (Zheng et al., 2004). The biological problem of viral emergence has not fundamentally changed, however the probability of occurrence of the risk is increasing owing to environmental change and higher environmental pressure.

## Anthropization and the accidental nature of disease emergence

**Figure 1:**
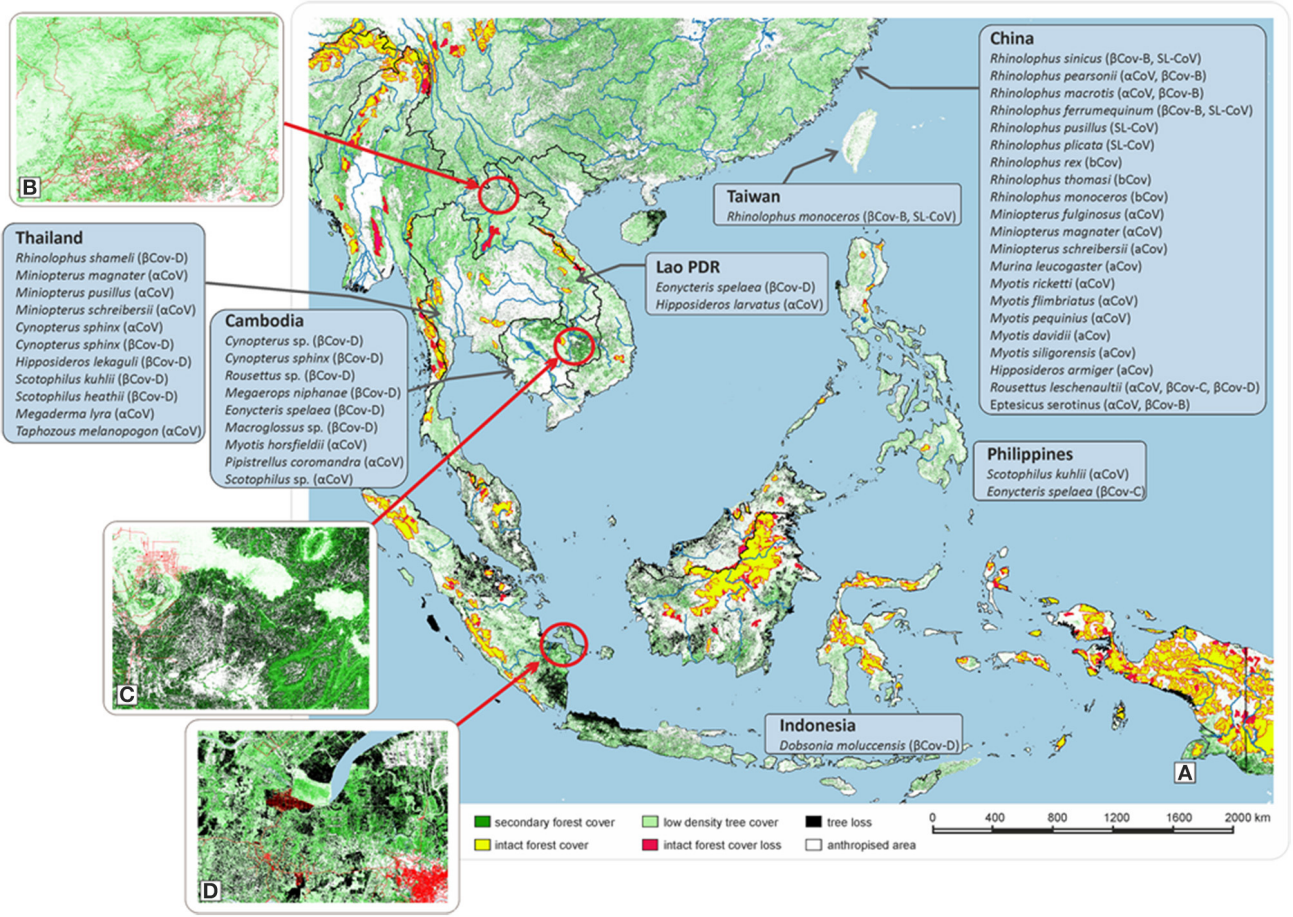
Evolution of the forest cover in Southeast Asia. **(A)** Changes in forest cover in Southeast Asia from 2000 to 2013. Time-series analysis of Landsat images with a resolution of 30 × 30 m. The nature of the vegetation cover (stability or change over a period of 13 years), is indicated by a color code: Yellow: Remaining primary forest (a primary forest is defined as a mosaic of forests and naturally treeless ecosystems within the zone of the current forest extent, which displays no remotely detected signs of human activity or habitat fragmentation and is large enough to maintain all native biological diversity). Red: Lost primary forest, Black: Tree loss, Green: Forest cover, Light green: Low-density tree cover, White: Anthropized area (cultivated land and settlements, including cities). The types of coronaviruses isolated and described in several countries in Asia are presented along with the bat species from which they have been isolated. **(B)** Example of forest cover loss in Lao PDR with evidence of a low-density tree cover. Land cover evolution from 2000 to 2013. Data obtained using a 30 × 30 m resolution (Black: Tree loss; green: Forest cover; light green: Low-density tree cover). **(C)** Example of forest cover loss in Cambodia where deforestation linked to wood trade and agriculture. Land cover evolution from 2000 to 2013. Data obtained using a 30 × 30 m resolution (Black: Tree loss; green: Forest cover; light green: Low-density tree cover). **(D)** Example of forest cover loss in Sumatra (Indonesia) where deforestation was linked to population growth and agricultural pressure. Land cover evolution from 2000 to 2013. Data obtained using a 30 × 30 m resolution (Black: Tree loss; green: Forest cover; light green: Low-density tree cover).

The “One Health” concept recognizes that human health is connected to animal health and to the environment. Southeast Asia (SEA) is the region in the world that has suffered the greatest rate of deforestation with a loss of 30% of forest surface over the last 40 years (Figure 1A). In Thailand, agricultural lands amounted to 23% in 1960 of total land area vs. 40% since 1985^1^ Similar trends were observed in other Southeast Asian countries^1^. In Cambodia, agricultural surfaces doubled from 15% in the 1980s up to 30% in 2000. An even higher increase was observed in Vietnam with an increase from 20% in 1990 to 35% nowadays. In Indonesia, the growth rate rose from 21% in the 1980s to 31.5% nowadays. Deforestation is currently linked to increased agricultural surfaces and poorly-managed urban growth (Figures 1B–D). Human population in SEA increased by 130 million between 2001 and 2011 and is expected to rise by almost 250 million by 2030^2^ This demographic growth generates pressure on land use, agricultural land and deforestation, with the most common activities being farming, logging, and hunting. For instance, in Sumatra (Figure 1D), an area deforested over the last 13 years was turned into a dynamically growing suburban zone with intensive farming. Owing to evolving land-use, bat populations are setting up in areas closer to human dwellings (Reuter et al., 2016). Anthropized rural environments are characterized by a wide diversity of landscapes comprising houses, barns, fields, orchards, and woods of differing density. The common belief is that deforestation and anthropization will lead to the disappearance of species. This is not always true and anthropized environments can provide an acceptable habitat for a large range of bat species, generating thus a higher diversity of bats and in turn of bat-borne viruses next to human dwellings (Plowright et al., 2015; Afelt et al., 2018). Anthropization generates a highly diverse environment in the vicinity of human populations, characterized by differing forest densities. Unlike natural environments which are highly selective, these altered landscapes are acceptable by a wide range of bat species, usually not encountered together. They can find there anthropized environmental niches compatible with their roosting and hunting needs (Walsh et al., 2017; Afelt et al., 2018). Furthermore, house lights attract a large number of insects at night, offering easy prey for insectivorous bats. Houses and barns offer shelter for cave-dwelling bats while orchards and fields attract frugivorous bats. This attractive effect of anthropized environments on bats with differing biological needs results in a higher concentration and biodiversity of bat-borne viruses (Han et al., 2015; Plowright et al., 2015; Reuter et al., 2016; Lacroix et al., 2017a,b; Walsh et al., 2017; Afelt et al., 2018). This increases the risk of transmission of viruses through direct contact, domestic animal infection, or contamination by urine or feces. CoVs being primarily agents of veterinary diseases, the risk of emergence of disease is as much on domestic animal diseases as on human diseases.

However, the emergence of a disease is impossible to predict. It is an accidental process, i.e., the occurrence of an extremely low probability event resulting from a stochastic combination of low probability independent events. If the exact time and nature of the emergence of a disease cannot be predicted, the increased probability of encounter and occurrence of an emergence-leading chain of events yielded by anthropized environments must be considered seriously. Until now, there is no evidence for CoV circulating in bats to be directly at the origin of infection in humans. The SARS-like bat CoV was transmitted to humans after having evolved in the Himalayan palm-civet (Song et al., 2005). MERS-like bat CoV, might have originated in vespertilionid bats and then evolved in dromedary prior to human transmission (Corman et al., 2016). The emergence of MERS cannot be attributed to deforestation but instead to the close vicinity of people and camels (Goldstein and Weiss, 2017). However, The MERS virus was found in *Taphozous* bats living in ruins and other domestic animals might have been involved (Smith and Wang, 2013). Outside CoVs, something similar happened in 1994 with the Hendra virus in Australia when the *Pteropus* bat-borne virus was transmitted to horses and from horses to humans, most likely though aerosols (Halpin et al., 2000).

## Bat-borne viruses: a historical foe with a bright future

Major human infections by bat-borne viruses have been documented quite recently, although they might have occurred earlier in history. CoVs were mostly associated with veterinary diseases, with livestock and pets acting as intermediate carriers for transfer to humans. About 4.4% of the rats sold in three live markets in the Mekong Delta region in Vietnam and 22% of the bats sampled in three bat farms carried CoV, which is a high level of animal contamination (Berto et al., 2017). Before SARS-CoV and MERS-CoV emerged in humans, the four known human CoVs (HCoV-HKU1, HCoV-229E, HCoV-NL63, and HCoV-OC43) had been reported as endemic and responsible for mild to moderate respiratory tract diseases during more than three decades. Evidence indicates that alpha CoVs from the bat *Hipposideros caffer ruber* shared common ancestors with human HCoV-229E (Pfefferle et al., 2009) and that a related virus infected captive alpacas (*Vicugna pacos*), while another related virus infected camels (Corman et al., 2016). Furthermore, HCoV-NL63, found in 9.3% of samples from people hospitalized for respiratory diseases displays sequence similarities with the bat (*Perimyotis subflavus*) CoV ARCoV.2, whereas HCoV-NL63 can replicate in cell lines derived from the lungs of tricolored bats (Huynh et al., 2012). MERS-CoV is closely related to both bat CoV HKU4 (found in *Tylonycteris* bats) and bat CoV HKU5 (found in *Pipistrellus* bats). Altogether, these data illustrate the complex dynamics of CoV circulation between bats and wild or domestic (bovine, pigs) animals prior to crossing to humans. The situation is quite different with the emergence of a novel pathogen within the immune-naive human population. In such a case, the risk of large epidemics is very high along with high mortality. Once adapted to humans, CoVs may evolve to develop a more efficient intra-species mode of transmission. During SARS outbreaks in Toronto and Taiwan, certain persons were very efficient at transmitting SARS-CoV and were named “Superspreaders” (McDonald et al., 2004). A total of 83.2% of the transmission events were epidemiologically linked to five “superspreaders,” all of whom had pneumonia diagnosed at the first medical consultation.

## CoVs and beyond

Unfortunately, the problem of bat-borne viruses is not restricted to CoVs. Among the 60 viral species reported to be associated with bats, 59 are RNA viruses which might possibly be responsible for emerging and re-emerging infectious diseases in humans (Brook and Dobson, 2015). However, bats are not necessarily involved in primary infection of humans. The main risk for emergence of diseases is directly linked to the development of anthropized environments and their attractiveness for different bat species. Several examples can be found in other viral families. The Hendra virus was detected in 1994 after the death of 30 horses and 1 man in Hendra, Australia. The most likely way of human contamination was aerosols from diseased horses which were initially contaminated by urine or amniotic liquid from *Pteropus* bats (Weatherman et al., 2017). The Nipah virus is another example of the combined effect of deforestation and attraction to anthropized environments. *Pteropus* bats affected by deforestation settled in barns where they transmitted the virus to pigs which in turn infected humans (Chadha et al., 2006). Human-to-human contamination also occurred (Anthony et al., 2017). Infection by the Nipah virus led to a mortality rate of up to 74% in humans (Lin et al., 2017). Lyssaviruses bring other examples of bat-borne viruses infecting wild and domestic mammals and humans and transmitted through bites. The best known virus from this family is rabies, but other lyssaviruses like Australian Bat Lyssavirus (ABLV), Lago virus or Duvenhage virus also represent a threat. ABLV and Duvenhage virus are examples of bat-borne viruses directly transmitted to humans by bats (Tignor et al., 1977; Hanna et al., 2000; Paweska et al., 2006). These events of direct transmission remain rare but they nevertheless stress the risk associated with a higher biodiversity of bats and a higher density of bat populations in close proximity to humans. Anthony and colleagues have estimated that there are at least 3,204 CoVs currently circulating in bats (Anthony et al., 2017). Whatever the accuracy of that prediction, it remains obvious that the risk for new viruses to emerge from bats is probably very high. By being one of the regions of the world where population growth is the strongest, where sanitary conditions remain poor and where the deforestation rate is the highest, SEA meets every condition to become the place of emergence or re-emergence of infectious diseases.

## Conclusions

A recent phylogenetic study has provided strong evidence that viruses isolated from bats in China are clustering by geographical location rather than by bat species, suggesting that high contact rates among specific bat species favor the spread of CoVs (Lin et al., 2017). It is believed that most CoVs, if not all, are also circulating in different mammal species originating from ancestral bat CoVs. Notably, only a small minority of the estimated 1,240 bat species has been tested for CoVs. It is likely that many more CoVs could be discovered in bats. Although 31% of bat-borne viruses are CoVs (Calisher et al., 2006), only 6% of all CoV sequences in GenBank are from bat CoVs. Even though the direct transmission of bat CoVs to humans has not been evidenced yet, the creation of conditions for more frequent encounters between bat CoVs, domestic animals and humans poses a significant threat for the future (Chan et al., 2013). Considering that the increasing impact of human activities on the ecosystems is unlikely to abate in SEA, it is necessary to increase CoV surveillance in wildlife, cattle, pets and humans to better understand the dynamics of interspecies transmission and improve risk assessment, early warning and intervention (Devaux, 2012). It will certainly be crucial to pay special attention to “superspreaders” who are very efficient at transmitting CoVs through exposure to respiratory droplets. The emergence of a disease is an accidental process and it is therefore impossible to predict the scenarios and dynamics of emerging infectious disease events. The attractive effect on bats of anthropized environments is a major risk factor in the emergence of novel bat-borne diseases in both humans and animals. Also, given the share of CoVs described in bats, i.e., 31%, the risk of newly emerging CoVs-associated diseases in the future should be considered seriously. If a priority is to discover therapeutic options and vaccines (Graham et al., 2013; Zumla et al., 2016), it is even more important to work on education and people awareness regarding risks associated with anthropized environments.

## Author contributions

CD, RF, and AA participated in all parts of the work and in all analyses and writing; AA did the spatial analysis and developed the maps. All authors read and approved the final manuscript.

### Conflict of interest statement

The authors declare that the research was conducted in the absence of any commercial or financial relationships that could be construed as a potential conflict of interest.

## Footnotes

^1^http://data.worldbank.org/indicator/AG.LND.AGRI.ZS

^2^http://data.worldbank.org/indicator/SP.POP.TOTL
